# WinHAP: An Efficient Haplotype Phasing Algorithm Based on Scalable Sliding Windows

**DOI:** 10.1371/journal.pone.0043163

**Published:** 2012-08-14

**Authors:** Yun Xu, Wenhua Cheng, Pengyu Nie, Fengfeng Zhou

**Affiliations:** 1 Department of Computer Science and Technology, University of Science and Technology of China, Hefei, Anhui, China; 2 Anhui Province-MOST Co-Key Laboratory of High Performance Computing and Its Application, University of Science and Technology of China, Hefei, Anhui, China; 3 Shenzhen Institutes of Advanced Technology and Key Laboratory of Health Informatics, Chinese Academy of Sciences, Shenzhen, Guangdong, China; Aarhus University, Denmark

## Abstract

Haplotype phasing represents an essential step in studying the association of genomic polymorphisms with complex genetic diseases, and in determining targets for drug designing. In recent years, huge amounts of genotype data are produced from the rapidly evolving high-throughput sequencing technologies, and the data volume challenges the community with more efficient haplotype phasing algorithms, in the senses of both running time and overall accuracy. 2SNP is one of the fastest haplotype phasing algorithms with comparable low error rates with the other algorithms. The most time-consuming step of 2SNP is the construction of a maximum spanning tree (MST) among all the heterozygous SNP pairs. We simplified this step by replacing the MST with the initial haplotypes of adjacent heterozygous SNP pairs. The multi-SNP haplotypes were estimated within a sliding window along the chromosomes. The comparative studies on four different-scale genotype datasets suggest that our algorithm WinHAP outperforms 2SNP and most of the other haplotype phasing algorithms in terms of both running speeds and overall accuracies. To facilitate the WinHAP’s application in more practical biological datasets, we released the software for free at: http://staff.ustc.edu.cn/~xuyun/winhap/index.htm.

## Introduction

Genomic variations among individuals of a population of the same species can be classified as two families based on their lengths, i.e. single nucleotide polymorphisms (SNPs) and genome structural variations (GSVs) [1], [2]. Such genomic variations are supposed to the driving force for almost all of the human phenotypes, including both non-lethal (e.g. earwax types induced by the genotypes in ABCC11 [3]) and lethal ones (e.g. chronic myelogenous leukemia induced by the Philadelphia chromosome fusion [4]). The association of each SNP with a genetic disease has been extensively studied, and promising results for Huntington’s disease [5] and Marfan syndrome [6] have already been widely used for clinical diagnosis. Many genetic diseases were much more complex and were known to be associated with more than one genomic variation, e.g. tumor [7]. However, the required running time of *k*-SNP association with a given phenotype increases exponentially with the number *k* of SNPs. The number of frequent SNPs in the human population is estimated to be about 10 million, which makes it impossible to solve the general *k*-SNP association problem within reasonable time [8].

The diploid human genome has two copies of each chromosome. It is costly and time consuming to experimentally derive the sequence of alleles in contiguous SNP sites along each copy of the diploid chromosomes, which is called a *haplotype* [9]. So the two nucleotides/alleles for one position in the chromosome are usually derived as an unordered pair, which is called a *genotype*. If these two alleles of a genotype are the same nucleotide, the pair is called *homozygous*, otherwise *heterozygous*. The *haplotype phasing problem* is to determine the haplotypes in a list of genomes/genotypes. The number of candidate haplotypes grows exponentially with the number of heterozygous SNPs in the population. So it’s essential to design approximate algorithms for the haplotype phasing problem. The problem is usually simplified to study the phenotype association of adjacent SNPs in a continuous chromosome region [10].

The existing methods for the haplotype phasing problem can be classified into three major categories. The first category of algorithms focuses on finding the exact solution using combinatorial optimization algorithms, e.g. graph realization algorithms [11], [12]. The second category applies statistical tests to estimate the haplotype frequencies [13]. And the third one proposes heuristic rules to find the suboptimal solutions instead of the optimal one [14].

A combinatorial algorithm phases the haplotypes satisfying a set of phasing principles, among which the maximum parsimony represents one of the most widely used rules. Although theoretically there may be an exponential number of candidate haplotypes for a given genotype with a given number of heterozygous SNPs, the number of possible haplotypes existing in the real population is always limited. Maximum parsimony principle has been justified by experimental results and shows that the smallest haplotype set which can resolve all genotypes is close to the real haplotypes [12]. Gusfield proposed an Integral Linear Programming based algorithm to resolve it [12]. Wang and Xu [15] applied the branch and bound algorithm for this problem, which significantly speeds up the problem resolving. However, the basis of most haplotype phasing algorithms adopted the maximum parsimony principle, which is shown to be NP-complete [9] and APX-hard even in very restricted cases [16]. Another major principle is the perfect phylogeny tree principle [11], which claims to reconstruct the resulting haplotypes of individual genotype data from a perfect phylogeny tree. This principle assumes that any SNP mutation happened just once in the human evolutionary history. A number of approximation algorithms have been designed based on the assumption of the perfect phylogeny tree principle [11], [17], but the strong assumption holds back its application in some practical cases. The combinatorial algorithm usually works fairly well on a small-scale genotype dataset. When the size of the dataset grows large, its running time or accuracy becomes unacceptable [18].

Statistical tests are also widely used to phase the genotypes. Statistical algorithms estimate the frequencies of all possible haplotypes and choose the most probable haplotype pairs as the final solution. Such algorithms test the haplotypes with the maximum likelihood using various statistical algorithms, such as expectation maximization [13] and Bayesian [19]. Other statistical algorithms employ much more complex statistical models, such as fastPhase [20] and HaploRec [21]. The problem quickly becomes intractable for the larger dataset size of dataset. In order to further reduce the computation time requirement, the partition-ligation (PL) strategy is applied in the algorithms, such as PLEM [22] and GERBIL [23]. The PL strategy focuses on the haplotypes of adjacent regional sites instead of the whole haplotypes. It works well on a larger dataset. Firstly the methods based on the PL strategy partition the candidate haplotypes into uniform blocks. However, many studies characterizing human haplotype structure have shown that the SNPs are grouped into ‘blocks’ of different size for the individuals [24], [25]. Zhao YZ and Xu Y [26] presented a more accurate algorithm using a reasonable block partition and ligation strategy according to the haplotype structure. Although most statistical phasing algorithms extend the usefulness with more data processing strategies, the haplotype phasing problem still requires too much computation power, representing the major hurdles for this problem.

A large number of genotypes were generated with the large-scale sequencing technologies. In order to handle populations with more SNPs, heuristic strategies of exact algorithm are usually proposed to speed up the phasing of the genotypes. Tininini [14] designed a new heuristic algorithm (implemented as a program called CollHaps) for haplotype phasing, which based on the maximum parsimony principle and the iterative application of collapse rules. The software enables the user to process large data sets and obtain very “parsimonious” solutions in short time.

In this study, we proposed a new haplotype phasing algorithm based on scalable sliding windows and parsimony principle, which not only maintains the similar speed with the 2SNP but also has a much higher accuracy. In the *first step*, the initial haplotypes of individual genotype dataset are obtained based on simplified 2SNP method. In the *second step*, the haplotypes will be improved by the scalable sliding windows if in which a type of haplotype pair occupies the majority. The scalable sliding window is composed of consecutive SNPs which contain heterozygous SNPs, homozygous SNPs or missing SNPs. In the *final step*, the haplotypes are iteratively decreased by restricting one recombination at most in two haplotypes of each genotype based on parsimony principle. The algorithm is implemented in a software package called WinHAP. We test WinHAP on four different-scales datasets of genotypes. The result shows that WinHAP not only runs very fast, but also has high accuracy. It outperforms most of the existing algorithms in terms of both speed and accuracy.

## Methods

### Datasets

Firstly, we compared the performances of WinHAP and the other Haplotype Phasing algorithms on the data of angiotensin converting enzymes (ACEs) [27]. An ACE catalyzes the conversion of angiotensin I to the physiologically active peptide angiotensin II, which controls systemic blood pressure and fluid-electrolyte balance [27]. This dataset consists of the genotypes of 11 unrelated individuals, and each genotype is 52 in length. The genotypes were resolved into 13 distinct haplotypes through experiments. This dataset is denoted as ACE.

We further tested all the algorithms with another dataset on the genetic risk factors of Crohn disease [25]. This dataset consists of 129 pedigrees (father, mother and child), each genotyped at 103 SNPs in the chromosome 5q31 region. The genotypes of another 129 children were selected as the control. The original version of the control dataset has 13,287 SNPs, including 3,873 (29%) heterozygous SNPs and 1334 (10%) missing SNPs. After pedigree resolving, the phase of 2714 heterozygous SNPs and 168 missing SNPs could be determined. These identified SNPs were used for the accuracy evaluation of various Haplotype Phasing algorithms. This dataset is denoted as 5q31.

We chose the Cystic Fibrosis Transmembrane-Conductance Regulator (CFTR) gene dataset [28] to evaluate the algorithms’ performance for the different numbers of genotypes in the same haplotype dataset. This dataset is denoted as CFTR.

We constructed a larger dataset to test the algorithms’ performance from the HapMap database [10]. We simulated genotypes based on 30 children’ data of USA Utah residents in the ENr113 region of chromosome band 4q26. A random sampling from the 30 genotypes produced 1000 genotypes with length 1393, which is denoted as HapMap.

### Related Works

Brinza and Zelikovsky proposed a very fast algorithm, 2SNP, for haplotype phasing only SNP pairs [29]. 2SNP starts by calculating the certainty weight of *cis*- or *trans*-phasing on every pair of heterozygous sites *i* and *j*, where *cis*-phasing reduces the haplotype 22 to 00 and 11, and *trans*-phasing reduces 22 to 01 and 10. The formula for calculating the weight is as follows.
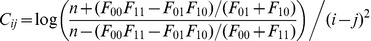
1where *n* is the number of input genotypes, and *F*_00_, *F*_01_, *F*_10_, *F*_11_ are the frequencies of haplotypes with the first and second binary indexes denoting alleles of the *i* ^th^ and *j* ^th^ SNPs. A complete graph is constructed with each node representing a heterozygous SNP and an edge weight being the certainty of *cis*- or *trans*-phasing on the two heterozygous SNPs corresponding to the two edge nodes. The maximum spanning tree can be determined and uniquely corresponds to the haplotype phasing of each genotype if they are *cis*- or *trans*-phased for all pairs of 2s. The algorithm can be finished within the time complexity *O*(*nm*(*n* + *m*)), where *n* and *m* are the numbers of genotypes and SNPs, respectively. It is fast enough for analyzing the high-throughput genotyping data, but its accuracy still remains to be improved.

The block structure of SNPs observed in the practical genotyping datasets is widely used to speed up the haplotype phasing algorithms and improve the phasing accuracy. The detailed patterns of SNP blocks were discovered by various linkage disequilibrium studies [30], [31], [32], [33]. In particular, Halperin and Eskin observed that the distribution of haplotypes in a block is uneven, and four main patterns of haplotypes constitute 97% of the haplotypes in each block [33]. The uneven distribution of haplotypes in a block is illustrated in Table 1 [33]. These frequent patterns are called the *common haplotypes*. The data suggests that a genotype matching a common haplotype has a much higher probability to be in the haplotype phasing solution. So it is reasonable to prioritize the common haplotypes in the haplotype phasing process.

**Table 1:** The uneven distribution of haplotypes in a block.

Haplotype	0/1 representation	Frequency
**CCGAT**	00000	66
**CTGAC**	01001	24
**ATACT**	11110	10
**CTGAT**	01000	6
**ATGAT**	11000	1
**ATGCC**	11011	1
**CCGAC**	00001	1

Most of the haplotype phasing algorithms follow the maximum parsimony principle, but its complexity of NP-completeness significantly slows down the algorithms’ running. An alternative way of finding a sub-optimal solution was adopted by all these algorithms, e.g. Hapinferx [34] and parsimonious tree-grow method [35].

### WinHAP Algorithm

In this section, firstly we will introduce the data structures of a haplotype and a genotype, and the haplotype phasing problem model. Then we describe in details the WinHAP algorithm, consisting of three major steps. In step 1, initial haplotypes are constructed by a simplified 2SNP-like algorithm. In step 2, the scalable sliding windows are used to correct the haplotype blocks which break apart the common haplotype pairs. In last step, the phasing solution is further refined by using a novel parsimonious strategy. We will analyze the computational complexity of the proposed algorithm at the end of this section.

The input to the haplotype phasing problem consists of *n* genotype vectors, each with *m* coordinates corresponding to *m* SNPs. Each SNP site is assumed to have at most two different alleles. The allele of a SNP site can be denoted by ‘0′ or ‘1′, where ‘0′ represents the major allele and ‘1′ the minor allele. A haplotype can be represented as a string of the alphabet {0, 1}. A genotype can be denoted as a string of the alphabet {0, 1, 2, ?}, where ‘0′ and ‘1′ represent the homozygous SNP {0, 0} and {1, 1}, respectively, ‘2′ is a heterozygous SNP {0, 1}, and ‘?’ is a missing SNP. The haplotype phasing problem can be defined formally as follows.

Definition 1 (*Haplotype Phasing Problem*): Given a genotype 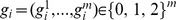, a pair of haplotypes 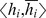 is the solution of *g_i_* when 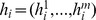 and 
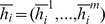
 satisfy the condition: for any 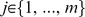, 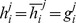 if 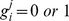, and 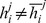 if 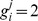.

When the above conditions hold, we say that *g_i_* can be phased to 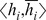, or ***g*** *_i_* can be resolved into 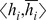, which is also denoted by 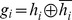. We will describe in details the three phasing steps to resolve the phasing problem on a given input set {***g***_1_, *…,* ***g*** *_n_*}.

#### Step 1: initial phasing based on simplified 2SNP algorithm

We first simplified the 2SNP algorithm to investigate a genotype with only two heterozygous SNPs, which can be resolved into the haplotypes 00/11 or 01/10. We used *cis*-phasing and *trans*-phasing to represent a pair of heterozygous SNPs resolved into 00/11 and 01/10, respectively, as similar to the 2SNP algorithm [29].

**Figure 1:**
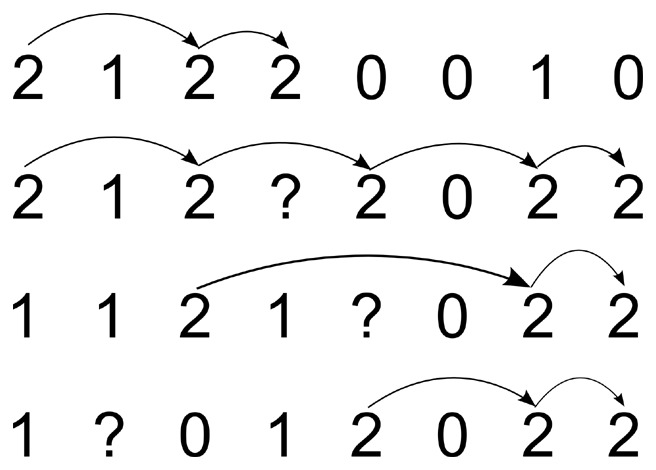
The initial phasing process in the first step.



We use the pairs of adjacent heterozygous SNPs to give the initial haplotypes, and then construct a linear tree, instead of the maximum spanning tree in the original version of 2SNP algorithm, to infer a solution to the haplotype phasing problem. For a *n×m* matrix of input genotypes, each row is an *m*-dimensional genotype 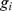 and its solution is haplotype 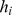 and 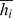. The *k* ^th^ heterozygous SNP of the *i* ^th^ genotype 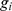
is denoted by 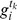. For each genotype, the *k* ^th^ heterozygous SNP 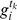 is paired with it’s next heterozygous SNP 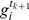. The phasing pattern (i.e. *cis*-phasing or *trans*-phasing) of each heterozygous SNP pair is determined according to the major haplotypes among the haplotypes 00/11 or 01/10. The phasing pattern of a heterozygous SNP pair is determined by the following formula (2). The first heterozygous SNP of each genotype is resolved into ‘0′ and ‘1′ (i.e. 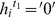, and 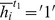). If the phasing pattern of a heterozygous SNP pair is determined, the other SNP site of this pair can be resolved uniquely. By repeating the procedures of pairing and phasing, the haplotypes of all the SNP sites can be resolved. Figure 1 shows this initial phasing process.

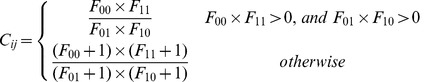
2where *F*_00_, *F*_11_, *F*_01_, *F*_10_ are the frequencies of haplotypes with the first and the second binary index, denoting alleles of the *i* ^th^ and *j* ^th^ SNP sites, respectively. Haplotype frequencies are determined based on all genotype frequencies except 22 and the genotypes that contains ‘?’.

#### Step 2: improvement of haplotypes using scalable sliding windows

Step 2 expands the haplotypes of adjacent SNP pairs into the results within scalable sliding windows. A *window* is a genotype segment with the same start and end SNP sites for all individuals within a population. These SNP sites could be heterozygous, homozygous or missing ones. In the process of moving window, each time the window slides to the right one position from the first SNP site on the genotypes. When the window moves to some SNP site, the window starts to the right extension that the length of the window changes from *l_min_* to *l_max_*, where *l_min_* and *l_max_* are the minimum and maximum values respectively. We called these removable and scalable windows as the *scalable sliding windows*.

Four main common patterns of haplotypes can explain over 97% cases within a block of SNP sites in a practical dataset [33]. Our algorithm tries to replace the phasing with common phasing patterns, within the scalable sliding windows detected in the above procedure. A genotype segment in a window is denoted by *g^w^*. The definition of the compatible haplotype pairs of *g^w^* are given below.

Definition 2 (*Compatible Haplotype Pair*): In a window, given a genotype segment *g^w^* and two haplotype segments 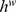 and 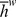. 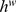 and 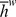 are called the compatible haplotype pair of *g^w^* if *g^w^* can be denoted by 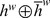.

The scalable sliding window starts from the first column of genotype matrix, and its length is set to *lmin*. The algorithm firstly calculates the frequencies of all possible haplotypes in the genotype window. Then all compatible haplotype pairs of each genotype are detected. For some genotype, if we find that a compatible haplotype pair occupies the majority of all compatible haplotypes pairs, the genotype is denoted as a block, and the resulting haplotypes of this genotype will be revised with the common haplotype patterns. The measurement of the *majority* is determined by the following formula.
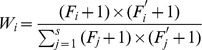
3where 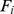 and 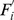 are the haplotype frequencies of the *i* ^th^ compatible haplotype pair of *g^w^*, and *s* is the number of all compatible haplotype pairs. When the maximum weight of all possible haplotype pairs is larger than threshold τ, the initial resulting haplotypes are replaced by the compatible haplotype pair with the maximum weight, otherwise keep the original haplotype pair unchanged. Each genotype segment in a window is processed in the same way. The windows with different lengths slide on the genotypes until the complete genotypes are corrected.

Let’s explain the above procedures with an example haplotype block from real data, as shown in Table 1. For a genotype segment 02002, two compatible haplotype pairs (00000 and 01001, 01000 and 00001) are detected. The weight of first haplotype pair (00000 and 01001) is 99%, whereas the other is 1%. If this genotype segment is resolved into 01000 and 00001 in the first step, our algorithm will replace them with 00000 and 01001. As observed in the real haplotype block data, the number of most common haplotype (e.g. 00000, 01001, 11110 or 01000) is more than 3 times larger than the number of another uncommon haplotype (e.g. 11000, 11011 or 00001). For this reason, we set the threshold *τ*  = 70% by default. The compatible haplotype pair with the maximum weight is called *the common haplotype pair*. Because the length of most blocks does not exceed 10 [26], the size of scalable sliding window is set in the range from 3 to 10 (i.e. *l_mix_*  = 3, *l_max_*  = 10).

One more thing, there is a case to be considered when the distance between neighbor heterozygous SNPs is larger than *l_max_*, because any window of the scalable sliding windows cannot cover this heterozygous SNP pair. For this case, we use the similarity to improve the results. A classical method for the similarity of two strings is Hamming distance. We assume these two heterozygous sites are *p_s_* and *p_e_*, and the genotype needing to be processed is the *i* ^th^ genotype. The *n* input genotypes are resolved into 2*n* haplotypes in previous processing, which are numbered from 1 to 2*n*. We use 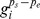 to denote the genotype segment (a sequence of homozygous SNPs) between *p_s_* and *p_e_* in the *i* ^th^ genotype, and use 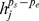 to denote the haplotype segment between *p_s_* and *p_e_* in the *j* ^th^ haplotype. 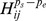 represents the Hamming distance between 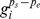 *and* 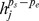, which is calculated by the following formulas.
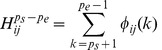
4where



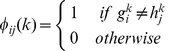
5The weight functions of *cis*-phasing and *trans*-phasing for the heterozygous SNPs pair are as follows.
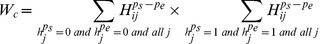
6

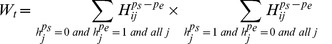
7


As the *cis*-phasing and *trans*-phasing of heterozygous SNP pair 22 are resolved into 00/11 and 01/10, respectively, the product of two sums is treated as the weight between them. Smaller Hamming distance represents greater similarity between two haplotypes. The phasing pattern (*i.e. cis*-phasing or *trans*-phasing) for this heterozygous SNP pair is updated by the one with smaller weight.

#### Step 3: refining haplotypes based on the maximum parsimony principle

**Figure 2:**
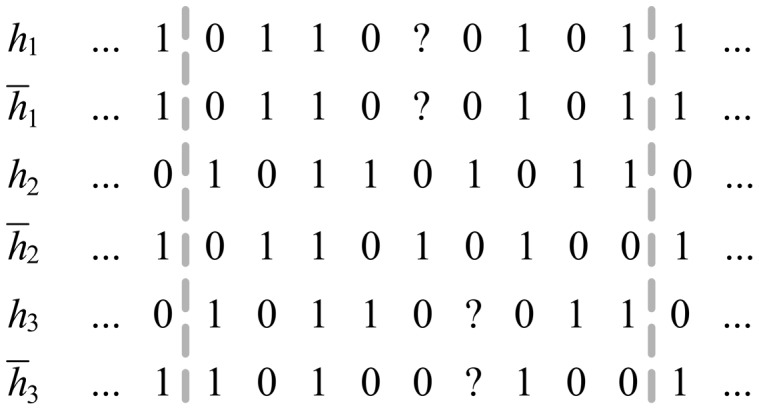
The recovery process of a missing allele on haplotype h1.

For the genotypes containing missing data, the resulting haplotypes also have missing alleles after the first two steps. The 2SNP algorithm recovers them with the corresponding values from the haplotype with the closest Hamming distance [29]. We define a window of length 9 for each missing allele, with the missing allele in the 5^th^ position. The missing data of a haplotype in the window is resolved by the similarity weights of the all other haplotypes with this haplotype. The computation of similarity weight between the haplotypes is the same as Step 2, where the similarity weights denotes the missing alleles being ‘0′ and ‘1′. Figure 2 gives an example of recovering missing data in haplotype *h_1_*.

The range between the dotted lines is the window to be investigated, as shown in Figure 2. Let *W*_0_ denote the weight of missing allele being ‘0′ in *h*_1_, and *W*_1_ denote the weight of missing allele being ‘1′. *W*_0_ is the sum of similarity weights of the other haplotypes where the corresponding missing position is ‘0′ (e.g. haplotypes *h*_2_, *h*_3_ and 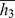), and *W*1 is the sum of similarity weights of the other haplotypes where the missing position is ‘1′ (i.e. haplotype 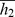). A similarity weight between *h*_1_ and *h*_2_ is calculated by formula (4), and added to *W*_0_. The missing alleles are finally determined by the allele (‘0′ or ‘1′) which has the smaller weight.

**Figure 3:**
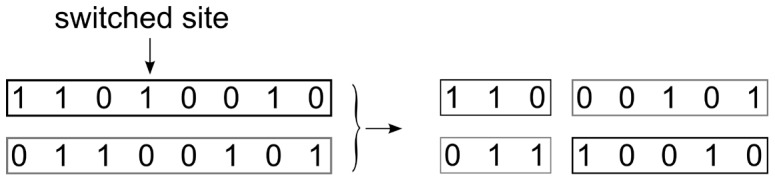
A recombination process with the 4th SNP as the switched site.

After the above processing, we can further optimize the haplotype solution set based on the maximum parsimony principle, minimizing the number different haplotypes. We used at most one recombination in two haplotypes of each genotype to make the current haplotype set smaller. The recombination process is illustrated in Figure 3. For example, haplotypes “11010010” and “01100101” are switched at the 4^th^ SNP site, so the resulting haplotypes are changed into “11000101” and “01110010”. The switched site must be heterozygous, and the recombination operation tries to decrease the number of different haplotypes. This investigation was iterated until the haplotype set size cannot be reduced.

### Time Complexity of WinHAP

Now, let us analyze the time complexity of our algorithm. In the first step, our algorithm uses the pairs of adjacent heterozygous SNPs to obtain the initial haplotypes, which takes *O*(*nm*) time since we have *n* genotypes each with *m* SNPs. In the second step, this algorithm improves the initial haplotypes using scalable sliding windows with length in the range from 3 to 10, which takes *O*(*n* ^2^ *m*) in time since every window should find all compatible haplotype pairs in *n* individuals. In the last step, our algorithm consists of two procedures. The recovery of missing data takes *O*(*n* ^2^ *m*) times since the process is similar to Step 2. The minimization of different haplotype number needs to investigate the recombination of each site in the worst case, which takes 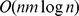 in time since every haplotype can be queried in the ordered haplotype list. Therefore our algorithm takes 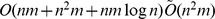 in total time, and it is on the same magnitude with the 2SNP algorithm.

### Measurement Criteria of Phasing Accuracy

As similar to most of the haplotype other phasing algorithms, we used the *individual error rate* (IER) [19] and the *switch error rate* (SER) [23] to evaluate the performance of phasing algorithm [36], [37]. The IER is defined as the percentage of individuals whose genotypes are incorrectly resolved. Generally, the IER value of phasing algorithms decreases with the increasing number of individuals, and increases with the increasing genotype length. The SER is defined as the ratio between the numbers of switch errors and all the heterozygous loci.

We compared our algorithm with six existing programs including ISHAPE [36], fastPhase [20], GERBIL [23], BBPLEM [26], Beagle [38] and 2SNP [29]. The program PHASE was not investigated because it didn’t produce the results within reasonable time. The program HaploRec [21] and CollHaps [14] was not tested because their current versions cannot handle the missing SNPs. All programs were run on the Linux operating system with a quad-core 3.1GHz CPU and 4GB memory.

## Results and Discussion

### Validation on Dataset ACE

We tested the performance of the algorithms on the dataset ACE, averaged over 100 independent runs. ISHAPE, GERBIL, and 2SNP were all run with the default settings. The parameter *K* (number of clusters) of fastPhase was set to 10 to reduce its running time. The buffer size of BBPLEM was set to 50 and the round of EM iteration was set to 20 which was the same with [26]. The parameter “*nsample*” of Beagle was set to 200 and we randomly generated the parameter “*seed*” in every independent running.

The performance of various phasing algorithms on ACE was shown in Table 2. WinHAP outperforms all the other algorithms in the error rates, with 0 in both IER and SER. Both 2SNP and GERBIL showed a slightly higher IER (0.091) and SER (0.005), which meant only one genotype was incorrectly resolved. All the other algorithms, such as ISHAPE, fastPhase, BBPLEM and Beagle, performed even worse accuracies on the ACE dataset. WinHAP also runs faster than all the other algorithms except 2SNP, with a slight increase in running time compared with 2SNP.

**Table 2:** Mean IER, SER and runtime of various phasing algorithms on the ACE dataset.

Software	IER	SER	Time (s)
**ISHAPE**	0.092	0.011	4.237
**fastPhase**	0.232	0.020	3.031
**GERBIL**	0.091^*^	0.005^*^	1.523
**BBPLEM**	0.178	0.020	0.039
**Beagle**	0.182	0.030	0.368
**2SNP**	0.091^*^	0.005^*^	**0.010**
**WinHAP**	**0.000**	**0.000**	0.019^*^

The results corresponding to the highest performance in each column are in bold. The results corresponding to the second-best performance in each column are attached an asterisk.

### Validation on Dataset 5q31

Because there were missing data in 5q31, the IER and SER were computed in the case of including and not including missing data, respectively. The IER1 is defined to exclude the missing data, and IER2 is defined to include the missing data. SER1 and SER2 are defined similarly. For this large dataset with 129 genotypes and 103 SNPs, the buffer size of BBPLEM was set to 100 and the iteration round of EM was set to 20, the same as [26]. The parameter “*nsample*” of BEAGLE was set to 25 to achieve phasing in short enough time. All the other parameters were set to the default values.

The seven algorithms were executed for 100 independent runs on the dataset 5q31, and the averaged performances are listed in Table 3. In most cases, WinHAP clearly outperforms the other algorithms in terms of both speed and accuracy. WinHAP achieved 0.326 in IER1, which is 1.5% lower than the second lowest value of ISHAPE. However, the running time of ISHAPE was more than 1000 times than WinHAP. As to IER2, fastPhase produced a minor improvement (0.77%) compared with WinHAP, but it runs for 149 times than WinHAP in time. As to SER1 and SER2, WinHAP achieved more than 3.8% improvement compared with the second best algorithm fastPhase, and its running time was more than 100 times less. Table 3 shows that WinHAP can produce comparable accuracy for missing data, within much shorter time.

**Table 3:** Mean IER, SER and runtime of various phasing algorithms on the 5q31 dataset with missing data.

Software	IER1	IER2	SER1	SER2	Time (s)
**ISHAPE**	0.331^*^	0.391	0.030	0.047	489.0
**fastPhase**	0.337	**0.385**	0.026^*^	0.041^*^	74.7
**GERBIL**	0.380	0.434	0.030	0.045	21.3
**BBPLEM**	0.341	0.388^*^	0.030	0.043	1.1
**Beagle**	0.343	0.400	0.028	0.043	1.8
**2SNP**	0.395	0.465	0.031	0.046	**0.4**
**WinHAP**	**0.326**	0.388^*^	**0.025**	**0.039**	0.5^*^

The results corresponding to the highest performance in each column are in bold. The results corresponding to the second-best performance in each column are attached an asterisk.

### Validation on Dataset CFTR

As similar in [14], [19], [39], we constructed a dataset of 57 haplotypes with no missing data from 94 experimentally identified disease haplotypes. In [19], [39], the haplotypes were randomly selected to form data sets of size 28. In our experiment, we used different size (28, 30, 35, 40, 45, 50) of haplotypes pairs set to form our genotype datasets, and for each size, 100 distinct data sets were generated. The buffer size of BBPLEM was set to 100 and the iteration round of EM was set to 20. The parameter “*nsample*” of BEAGLE was set to 25. All the other parameters were set to the default values.

Table 4 gives the accuracies and running times of the seven algorithms. For the dataset CFTR, ISHAPE produced the minimum IER and SER, but it runs for very long time on these datasets. The fastPhase also outperforms WinHAP in terms of IER and SER, but its running time is similar to ISHAPE. The BBPLEM gave the excellent accuracy with much less time on this dataset. When N is 40, 45 and 50, the IER and SER of BBPLEM are second lowest. Though the algorithm WinHAP proposed in this paper had higher IER and SER for different sizes, the running speed is almost the fastest. It’s highly significant that WinHAP produced an average 28.5% and 28% improvement in IER and SER for different sizes compared with 2SNP, respectivity. The other algorithms, such as GERBIL and Beagle, gave the higher IER and SER compared with WinHAP, and they run for 2 times than WinHAP in time.

**Table 4:** Mean IER, SER and runtime of various phasing algorithms on the CFTR dataset.

Software	N = 28			N = 30			N = 35		
	IER	SER	Time (s)	IER	SER	Time (s)	IER	SER	Time (s)
**ISHAPE**	**0.221**	**0.044**	3.90	**0.214**	**0.045**	4.14	0.191^*^	0.038^*^	4.73
**fastPhase**	0.252^*^	0.049^*^	3.73	0.246	0.049^*^	4.03	0.231	0.045	4.70
**GERBIL**	0.387	0.085	0.36	0.367	0.085	0.39	0.349	0.078	0.55
**BBPLEM**	0.261	0.054	0.02^*^	0.243^*^	0.051	0.02*	**0.182**	**0.037**	0.02^*^
**Beagle**	0.381	0.074	0.38	0.378	0.075	0.40	0.333	0.064	0.43
**2SNP**	0.423	0.089	**0.01**	0.414	0.087	**0.01**	0.417	0.083	**0.01**
**WinHAP**	0.316	0.065	**0.01**	0.312	0.066	**0.01**	0.301	0.061	**0.01**
	**N = 40**			**N = 45**			**N = 50**		
**ISHAPE**	**0.162**	**0.031**	5.67	**0.152**	**0.029**	6.34	**0.146**	**0.027**	7.31
**fastPhase**	0.214	0.040	5.38	0.214	0.039	6.00	0.208	0.039	6.68
**GERBIL**	0.324	0.070	0.70	0.334	0.072	0.86	0.325	0.069	0.95
**BBPLEM**	0.182^*^	0.037^*^	0.02^*^	0.176^*^	0.036^*^	0.02^*^	0.178^*^	0.036^*^	0.02^*^
**Beagle**	0.304	0.055	0.05	0.276	0.050	0.48	0.272	0.048	0.50
**2SNP**	0.396	0.079	**0.01**	0.388	0.077	**0.01**	0.405	0.080	**0.01**
**WinHAP**	0.285	0.056	**0.01**	0.260	0.053	0.02^*^	0.275	0.056	0.02^*^

The results corresponding to the highest performance in each column are in bold. The results corresponding to the second-best performance in each column are attached an asterisk.

### Validation on Dataset HapMap

For the largest dataset HapMap, the buffer size of BBPLEM was set to 100 and the interation round of EM was set to 20. The parameter “*nsample*” of BEAGLE was set to 25. All the other parameters were set to the default values.

For the large dataset HapMap, ISHAPE, fastPhase and GERBIL failed to gave a solution in 2 hours. Beagle can handle the genotype set in 55.5 seconds, but its IER and SER were the highest among all phasing algorithms. The algorithm BBPLEM had relatively low SER, but the running time is two times longer than Beagle. WinHAP and 2SNP outperform all the other algorithms in terms of both speed and accuracy. In particular, 37% improvement in SER was produced by WinHAP compared with 2SNP. WinHAP runs for minor longer time with significant improvement in accuracy, compared with 2SNP, as shown in Table 5. This showed that WinHAP could also deal with high-throughput genotype data with the significantly improved accuracy, compared with the fastest algorithm 2SNP.

**Table 5:** Mean IER, SER and runtime of various phasing algorithms on the HapMap dataset with no missing data.

Software	IER	SER	Time (s)
**ISHAPE**	–	–	No solution
**fastPhase**	–	–	No solution
**GERBIL**	–	–	No solution
**BBPLEM**	0.992^*^	0.027	126.1
**Beagle**	0.999	0.128	55.5
**2SNP**	0.999	0.024^*^	**38.6**
**WinHAP**	**0.965**	**0.015**	41.1^*^

The results corresponding to the highest performance in each column are in bold. The results corresponding to the second-best performance in each column are attached an asterisk.

## Conclusions

Huge amount of genotype data was being generated with the emerging large-scale sequencing technologies, at the increasing speeds. The development of a fast and more accurate haplotype phasing algorithm is necessary to meet the needs of handling massive genotype datasets. In this study, we proposed a fast haplotype phasing algorithms based on scalable sliding windows. The algorithm has better or comparable performance in both speed and accuracy in almost all cases, compared with the six other algorithms. We are working on both further improving the algorithm and applying it to study the haplotype phasing problem on our leukemia project.
